# New Sesquiterpenoids from *Ambrosia artemisiifolia* L.

**DOI:** 10.3390/molecules20034450

**Published:** 2015-03-10

**Authors:** Wenbing Ding, Rui Huang, Zhongshi Zhou, Youzhi Li

**Affiliations:** 1 Hunan Provincial Engineering & Technology Research Center for Biopesticide and Formulation Processing, Hunan Agricultural University, Changsha 410128, China; E-Mails: dingwenb119@hunau.edu.cn (W.D.); huangrui20082222@163.com (R.H.); 2 National Research Center of Engineering & Technology for Utilization of Botanical Functional Ingredients, Hunan Agricultural University, Changsha 410128, China; 3 State Key Laboratory for Biology of Plant Diseases and Insect Pests, Institute of Plant Protection, Chinese Academy of Agricultural Sciences, Beijing 100193, China; E-Mail: zhouzhongshi@caas.cn

**Keywords:** *Ambrosia artemisiifolia* L., sesquiterpenoid, sesquiterpene glucoside

## Abstract

A new pseudoguaianolide **1** and two new guaiane-type sesquiterpene glucosides **2** and **3**, were isolated from the aerial parts of *Ambrosia artemisiifolia* L together with two known sesquiterpene dilactones **4** and **5**. The new compounds were determined on the basis of spectroscopic and chemical methods to be 3β-acetoxy-4β-hydroxy-1α,7α, 10β,11αH-pseudoguaia-12,8β-olide (**1**), 1β,7β,9β,10β,13αH-guaia-4(5)-en-12,6β-olide 9-*O*-β-d-glucoside (**2**) and 4β-hydroxy-1α,5α,7α,9αH-guaia-10(14),11(13)-dien-12-acid 9-*O*-β-d-glucoside (**3**). The isolated compounds were evaluated for cytotoxicity against human promyelocytic leukemia HL-60 cell lines *in vitro*, but were all inactive.

## 1. Introduction

*Ambrosia artemisiifolia* L. (Asteraceae), an invasive alien plant species in China, is nowadays widespread in an area ranging from Guangdong Province in the south to Heilongjiang Province in the north [1]. This species is considered as a harmful weed capable of quickly colonizing both agricultural and urban areas with competitive seeds, and its pollen can induce serious allergic disorders during a certain period of its development [2]. However, many investigations into the composition and properties of *A. artemisiifolia* indicate that this species can serve a valuable source of biologically active substances. The extracts of this plant showed various bioactivities, such as molluscicidal [3], plant growth inhibitory [4], anti-inflammatory [5], antithrombin [6], antibacterial [7], insecticidal [8], hepatoprotective and hypolipemic activities [9]. On the other hand, the main chemical components of this species were identified as sesquiterpene lactones, which account for the antihelminthic, cardiotonic, antiinflammatory, analgesic, sedative (calming), antimalarial, anti-tumor, and other types of pharmacological activity [10,11]. As part of an ongoing search for novel bioactive compounds, we studied *Ambrosia artemisiifolia* L. growing in Hunan Province of China. Herein, this paper reports the isolation and identification of three new sesquiterpenoids from the aerial parts of the plant.

## 2. Results and Discussion

**Figure 1:**
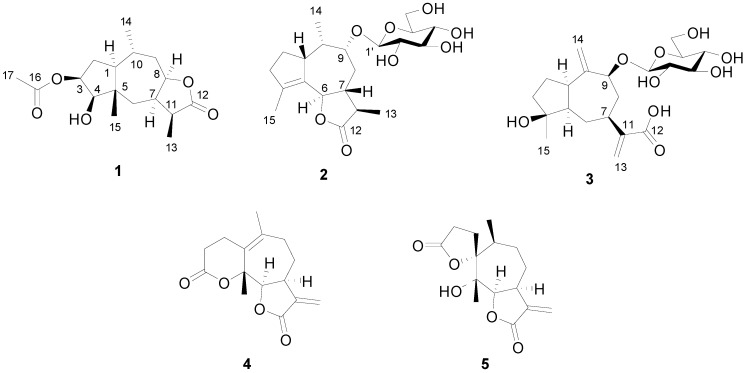
Structures of compounds **1**–**5** isolated from *A. artemisiifolia*.

The dried aerial parts of *A. artemisiifolia* were extracted three times with MeOH at room temperature. The MeOH extract residue was suspended in water and then partitioned successively with petroleum ether and EtOAc. Column chromatography of the EtOAc-soluble fraction yielded three new compounds **1**–**3** and two known compounds, psilostachyin B (**4**) [12] and psilostachyin (**5**) [13] (Figure 1).

**Figure 2:**
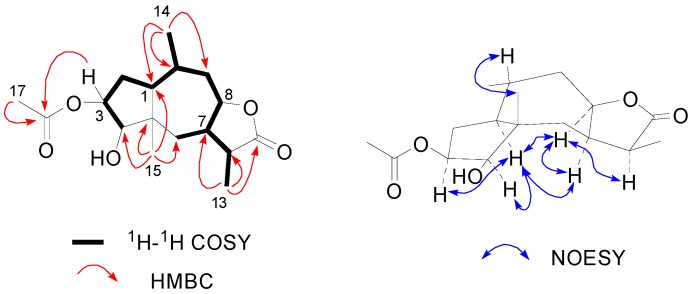
Key ^1^H-^1^H COSY, HMBC and NOESY correlations of **1**.

Compound **1** was obtained as white amorphous powder, and the molecular formula was assigned as C_1__7_H_2__6_O_5_ from its HRESIMS (*m/z* 311.1852 [M+H]^+^) and NMR data. The ^1^H-NMR spectrum displayed readily recognizable signals for four methyl groups [δ_H_ 1.18 (3H, s, H-15), 2.04 (3H, s, H-17), 0.90 (3H, d, *J =* 7.0 Hz, H-14) and 1.02 (3H, d, *J =* 7.4 Hz, H-13)] (Table 1). The ^13^C-NMR and DEPT spectra of **1** exhibited 17 carbons corresponding to four methyls, three methylenes, seven methines (including three oxy-methine carbons), a quaternary carbon, and two quaternary carbonyl groups (Table 1). The structure of **1** was deduced based on ^1^H-^1^H COSY, HSQC, and HMBC techniques using those methyl groups as starting points. The HMBC correlations from the H-atoms of the four methyl groups to corresponding C-atoms [H_3_-17 (δ_H_ 2.04) to C-16; H_3_-14 (δ_H_ 0.90) to C-1, C-9 and C-10; H_3_-13 (δ_H_ 1.02) to C-7, C-11 and C-12; H_3_-15 (δ_H_ 1.18) to C-1, C-4, C-5 and C-6) (Table 1)] established a typical acetyl moiety (δ_H_ 2.04, δ_C_ 169.8 and 20.3) and three fragments as ^14^CH_3_-^1^^0^CH(^1^CH)-^9^CH-, ^13^CH_3_-^11^CH (^7^CH)-^12^COOH, and ^15^CH_3_-^5^C(^4^CH/^1^CH)-^6^CH_2_- (Figure 2). Those fragments were further linked to form a 5/7-membered fused-ring structure based on the analysis of the ^1^H-^1^H-COSY correlations (Figure 2). Considering the molecular formula C_1__7_H_26_O_5_ and the presence of two C=O bonds, **1** was suggested to be an acetylated derivative of pseudoguaianolide [14]. The lactone ring was formed between C-12 and C-8, which deduced from the low-field chemical shift of C-12 (δ_C_ 177.8) and C-8 (δ_H_ 4.58 and δ_C_ 79.7) [15]. While the acetyl moiety was found to be attached to C-3 via an ester linkage from long-range correlation of H-3 [δ 5.45 (ddd, *J =* 8.1, 7.5, 5.0 Hz)] with C-16 (δ_C_ 169.8) observed in the HMBC spectrum (Figure 2). Thus the planar structure of **1** was deduced as 3-acetoxy-4-hydroxy-pseudoguaia-12,8-olide. The relative stereochemistry of **1** was assigned by analyses of the NOESY spectrum and the proton coupling patterns (Table 1). The key NOE correlation observed between H-7 and H-8, as well as the large coupling constant measured for H-8 (8.6 Hz), indicating the presence of a *cis*-fused lactone ring [16]. While the NOE correlation was absent between H-1 and H_3_-15 suggesting a typical *trans*-fused 5/7-membered ring, a model of this molecule indicated that H-1 was α-orientation [14]. Meanwhile, the correlations of H-1 with H-3, H-4 and H-7, and H-7 with H-8 and H-11 revealed that all of these protons (H-1, H-3, H-4, H-7 and H-11) were cofacial and were arbitrarily assigned α-oriented. The β-orientation of H-10 was revealed by the coupling constant measured H-1 (*J*_H-__1/H-10_ = 13.6 Hz), and corroborated by an NOE between H-10 and H_3_-15. Finally, compound **1** was established as 3β-acetoxy-4β-hydroxy-1α,7α,10β,11αH-pseudoguaia-12,8β-olide.

**Table 1:** NMR assignments of **1** by DEPT, HSQC, HMBC, and NOESY experiments in C_5_D_5_N *^a^*.

Position	δ_H_ (*J* in Hz)	δ_C_, Mult.	HMBC	NOESY
1	1.51, ddd (13.6, 5.6, 5.6)	39.0 CH	C-2, C-4, C-5, C-10, C-14,C-15	H-3, H-4, H-7
2	Hα: 1.82, m	31.5 CH_2_	C-1, C-3	
	Hβ: 2.01, m		C-1, C-3	
3	5.45, ddd (8.1, 7.5, 5.0)	71.0 CH	C-1, C-4, C-5, C-16	Hα-2, Hβ-2, H-4
4	3.64, d (7.5)	75.9 CH	C-3, C-5, C-6, C-15	H-1, H-3, H-7
5	–	43.3 qC		
6	Hα: 1.94, br d (14.3)	30.5 CH_2_	C-5, C-7, C-15	H-4, H-7
	Hβ: 1.16, overlapped			Hβ-9
7	2.51, m	36.7 CH	C-5, C-6, C-11, C12	H-1, H-4, H-8, H-11
8	4.58, ddd (13.2, 8.6, 2.8)	79.7 CH	C-7	H-7, Hα-9, Hβ-9, H-11
9	Hα: 2.07, m	36.4 CH_2_	C-1, C-7, C-8, C-10, C-14	
	Hβ: 1.66, br d (12.7)		C-1, C-7, C-8, C-10	Hβ-6, H-14, H-15,
10	1.80, m	28.5 CH	C-1, C-5, C-14	
11	2.94, m	37.5 CH	C-6, C-7, C-12, C-13	H-7, H-8, H-13
12	–	177.8 qC		
13	1.02, d (7.4)	9.7 CH_3_	C-7, C-11, C-12	Hβ-6, H-7, H-11
14	0.90, d (7.0)	16.4 CH_3_	C-1, C9, C-10	Hα-9
15	1.18, s	17.5 CH_3_	C-1, C-4, C-5, C-6	Hβ-9, H-10
16	–	169.8 qC		
17	2.04, s	20.3 CH_3_	C-16	H-3

Note: *^a^* ^1^H (400 MHz) and ^13^C (100 MHz) NMR; δ in ppm.

**Figure 3:**
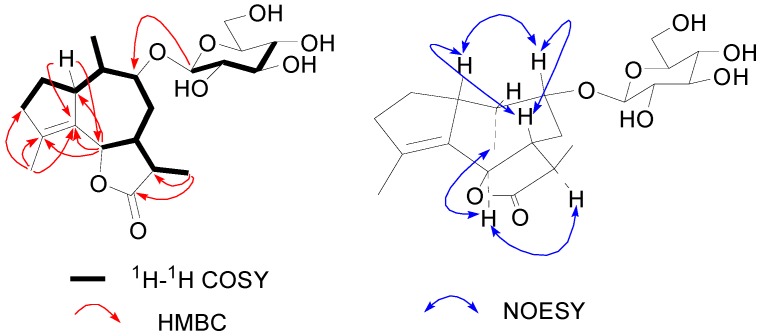
Key ^1^H-^1^H COSY, HMBC and NOESY correlations of **2**.

Compound **2** has the molecular formula C_2__1_H_32_O_8_ according to its HRESIMS and ^13^C-NMR data. ^1^H-, ^13^C-NMR and DEPT spectra revealed that **2** contained a typical β-d-glucopyranosyl [δ_H_ 4.38 (d, *J* = 7.8, H-1'); δ_C_ 102.5, 75.2, 77.9, 71.8, 78.2, 62.8], which was confirmed by acid hydrolysis and then co-chromatography with an authentic sample. Besides, the remaining 15 carbon signals, which belong to the aglycone, were attributable to three methyls, three methylenes, six methines (including two oxy-methines), two olefinic quaternary carbons, and a quaternary carbonyl group (Table 2). To deduce the structure of the aglycone and the glycosidic connection, a complete ^1^H and ^13^C-NMR spectral assignment was carried out using a combination of DEPT, HSQC, ^1^H-^1^H COSY, HMBC and NOESY experiments. The ^1^H-^1^H COSY spectrum of **2** revealed a fragment with eleven carbons [-^3^CH_2_-^2^CH_2_-^1^CH-^1^^0^CH(^14^CH_3_)-^9^CH(OR)-^8^CH_2_-^7^CH(^6^CHOR)-^11^CH-^13^CH_3_] which was corroborated by HMBC correlations (Figure 3). The fragment was further connected by HMBC spectrum, the key HMBC correlations from H-1 to C-4, C-5 and C-6, from H-6 to C-1, C-4 and C-5, from H-13 to C-11, C-12 and C-7, and from H-15 to C-3, C-4 and C-5 indicated a sesquiterpene skeleton with a 5/7-membered fused-ring, which belong to that kind of guaiane-type sesquiterpenoid (Figure 3) [17,18]. In addition, the oxy-methine carbon δ_C_ 82.6 (C-9) showed a long-range correlation with the anomeric proton of glucose at δ_H_ 4.38 (d, *J* = 7.8 Hz, H-1'), indicating that the hydroxyl group at C-9 is glucosylated. The low-field chemical shift of δ_C_ 181.2 (C-12) and δ_C_ 83.3 (C-6), as well as the molecular formula C_2__1_H_32_O_8_ revealed that a lactone ring was formed between C-12 and C-6 [18,19]. Therefore, the planar structure of compound **2** was determined as guaia-4(5)-en-12,6-olide 9-*O*-β-d-glucoside. In the NOESY spectrum, there were no NOE correlations between H-6 and H-7, as well as the large values of the constants of *J*_6,7_ and *J*_7,11_ (10–11 Hz), suggesting the presence of a *trans*-fused lactone ring [19]. Moreover, the correlations of H-7 with H-1 and H-9, and H-6 with H-11 and H_3_-14 revealed that protons H-7, H-1 and H-9 were cofacial, while H-6, H-11 and H_3_-14 were on the opposite side. Assume the usual α-configuration for the isopropyl at C-7 [19], the stereochemistry of **2** was established as 1β,7β,9β,10β,13αH-guaia-4(5)-en-12,6β-olide 9-*O*-β-d-glucoside.

**Table 2:** ^1^H- (400 MHz) and ^13^C- (100 MHz) NMR spectral data of compounds **2** and **3**.

Position	2 *^a^*		3 *^b^*	
	δ_H_ (*J* in Hz)	δ_C_, Mult.	δ_H_ (*J* in Hz)	δ_C_, Mult.
1	3.09, m	49.9 CH	3.24, m	45.4 CH
2	Hα: 1.56, m; Hβ: 2.10, overlapped	29.4 CH_2_	1.96, m	27.0 CH_2_
3	2.36, m	39.4 CH_2_	Hβ: 1.77, m; Hα: 1.98, m	40.9 CH_2_
4	–	131.6 qC	–	80.7 qC
5	–	142.5 qC	2.38, br t (10.8)	54.2 CH
6	4.80, d (11.5)	83.3 CH	Hβ: 1.39, ddd (12.2, 11.0, 10.8); Hα: 2.01, br d (12.2)	33.4 CH_2_
7	1.89, ddd (11.5, 11.6, 11.6)	46.8 CH	2.92, br t (11.0)	39.8 CH
8	Hα: 1.67, m; Hβ: 2.14, overlapped	32.2 CH	Hα: 1.88, m; Hβ: 2.62, br d (11.8)	45.3 CH_2_
9	3.88, m	82.6 CH	4.79, dd (11.0, 3.0)	79.5 CH
10	2.11, overlapped	41.3 CH	–	152.5 qC
11	2.34, m	42.5 CH	–	149.0 qC
12	–	181.2 qC	–	169.8 qC
13	1.18, d (6.9)	12.5 CH_3_	5.55, br s; 6.42, br s	121.9 CH_2_
14	0.80, d (7.0)	8.1 CH_3_	5.27, br s; 6.18, br s	110.1 CH_2_
15	1.81, s	15.5 CH_3_	1.32, s	24.9 CH_3_
Glc-1'	4.38, d (7.8)	102.5 CH	5.06, d (7.7)	101.1 CH
2'	3.15, dd (8.0, 7.8)	75.2 CH	4.11, dd (8.2, 7.7)	75.8 CH
3'	3.35, m	77.9 CH	4.23, m	78.7 CH
4'	3.31, m	71.8 CH	4.26, m	71.9 CH
5'	3.27, m	78.2 CH	3.88, m	78.8 CH
6'	3.66, dd (11.6, 5.2); 3.88, br d (11.6)	62.8 CH_2_	4.36, dd (11.9, 5.2); 4.51, dd (11.9, 2.2)	62.9 CH_2_

Notes: *^a^* measured in CD_3_OD, *^b^* measured in C_5_D_5_N; δ in ppm.

**Figure 4:**
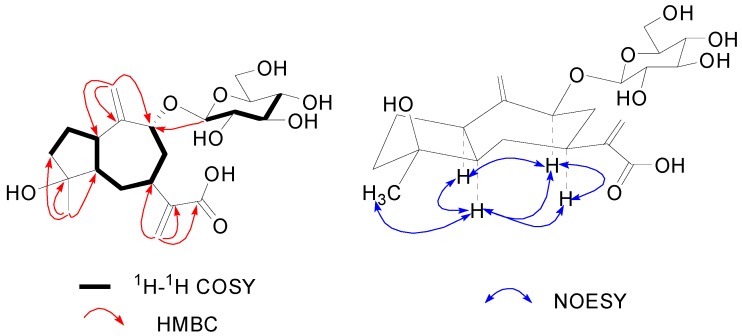
Key ^1^H-^1^H COSY, HMBC and NOESY correlations of **3**.

Compound **3** was obtained as a colorless gum. The molecular formula was shown to be C_2__1_H_32_O_9_ by its HRESIMS and ^13^C-NMR data. The structure of **3** contained a β-d-glucopyranosyl, which was confirmed by methods previously described in compound **2**. Moreover, two pair of exomethylene singlets at δ_H_ 5.55 /6.42 (br s, H_2_-13) and 5.27 /6.18 (br s, H_2_-14), along with a methyl singlets δ_H_ 1.32 (s, H_3_-15) was clearly observed in ^1^H-NMR spectrum. The ^13^C-NMR and DEPT spectra (Table 2) showed a total of 21 carbon signals, among which, 15 were ascribable to the aglycone. The structure of the aglycone and the glycosidic connection were carried out by 2D-NMR methods (HSQC, ^1^H-^1^H COSY, HMBC and NOESY experiments). The HMBC correlations from the H-atoms of the exomethylenes and methyl groups to corresponding C-atoms revealed three C_4_ units as ^13^CH_2_=^11^C (^7^CH)-^12^COOH, ^14^CH_2_=^1^^0^C(^1^CH)-^9^CH-, and ^15^CH_3_-^4^C(^3^CH_2_)-^5^CH-, respectively. Those C_4_ units in combination with a C_8_ unit -^3^CH_2_-^2^CH_2_-^1^CH-^5^CH-^6^CH_2_-^7^CH-^8^CH_2_-^9^CHOR deduced from ^1^H-^1^H COSY correlations (Figure 4), established the structure of the aglycone as 4,9-dihydroxyguaia-10(14),11(13)-dien-12-acid, which was good agree with the formula C_2__1_H_32_O_9_. Moreover, an informative correlation was also observed between the anomeric proton signal at δ_H_ 5.06 (d, *J* = 7.7 Hz, H-1') and a methine carbon signal at δ_C_ 79.5 (C-9) in the HMBC spectrum (Figure 4), implying that the sugar moiety was linked at the C-9 position. Therefore, the planar structure of **3** was established as 4-hydroxyguaia-10(14),11(13)-dien-12-acid 9-*O*-β-d-glucoside. The key NOESY correlations of H-1 with H-5 and H-9, and H-5 with H-7, H-9 and H_3_-15 showed that all of those protons (H-1, H-5, H-7, H-9 and H_3_-15) were cofacial. Meanwhile, the coupling constants of H-9 (δ_H_ 4.79, dd, *J* = 11.0, 3.0 Hz) revealed its α-oriented position. Thus compound **3** was determined to be 4β-hydroxy-1α,5α,7α,9αH-guaia-10(14),11(13)-dien-12-acid 9-*O*-β-d-glucoside.

The cytotoxicity of compounds **1**–**5** were tested against human promyelocytic leukemia HL-60 cell by the MTT method *in vitro*. The results revealed that all compounds were inactive (LC_50_ > 100 μM).

## 3. Experimental Section 

### 3.1. General

Optical rotations were measured on WZZ2B automatic polarimeter (Precision Instrument Co., Shanghai, China); melting points were obtained on a SGW X-4 micromelting point apparatus (INESA Physico Optical Instrument Co., Ltd, Shanghai, China). The 1D- and 2D-NMR spectra were measured with a Bruker DRX-400 instrument (Bruker BioS-pin GmbH Company, Rheinstetten, Germany) with TMS as internal standard. ESIMS data were recorded on an API QSTAR mass spectrometer (Applied Biosystem/MSD Sciex, Concord, ON, Canada). Column chromatography was performed on silica gel 60 (200–300 mesh, Qingdao Marine Chemical Ltd, Qingdao, China), Sephadex LH-20 (GE Healthcare, Uppsala, Sweden) and Develosil ODS (50 μm, Nomura Chemical Co. Ltd., Osaka, Japan). Preparative HPLC was performed on a Waters 1525 Binary HPLC pump and a Waters 2414 refractive index detector (Waters Corp, Millipore, Milford, MA, USA) using a YMC-Pack ODS-A column (250 mm × 10 mm I.D.; S-5 μm, 12 nm).

### 3.2. Plant Material

The aerial parts of *A. artemisiifolia* were collected from Miluo, Hunan Province, P. R. China, in August 2012, and identified by Prof. Zhongshi Zhou, Institute of Plant Protection, Chinese Academy of Agricultural Sciences. The voucher specimen (No. 20120821) was deposited in Hunan Agricultural University.

### 3.3. Extraction and Isolation 

Dried aerial parts of *A. artemisiifolia* (10.0 kg) were powdered and extracted three times with MeOH (95% v/v) at room temperature, then concentrated under reduced pressure to obtain a crude residue (0.5 kg). The residue was further suspended in H_2_O (2 L) and extracted with petroleum ether (PE) and EtOAc successively, to yield a PE-soluble fraction (90.0 g), an EtOAc-soluble fraction (90.0 g). The EtOAc-soluble fraction was subjected to silica gel column chromatography (CC) (100–200 mesh) with elution of CHCl_3_–MeOH (100:0 → 60:40, v/v) to give six fractions (Fr. C_1_–C_6_). The Fr. C_2_ was further separated by ODS-C_18_ CC (MeOH–H_2_O 30:70 → 70:30, v/v) to produce seven sub-fractions (C_2_-1–C_2_-7), then sub-fraction C_2_-3 was further purified by Sephadex LH-20 column (MeOH) and normal silica gel CC (petroleum ether–EtOAc, 9:1) to yield colourless crystal **4** (100.0 mg) and **5** (30.0 mg). Similarly, Fractions C_3_ (6.0 g) and C_5_ (8.3 g) were fractionated by an ODS-C_18_ column with elution of MeOH–H_2_O (30:70 → 70:30, v/v), respectively. Compound **1** (60.0 mg) was obtained from sub-fraction C_3_-5 by Sephadex LH-20 column (MeOH) and semi-preparative HPLC chromatography (MeOH-H_2_O 48%, v/v, flow rate 3 mL/min, *t*_R_ = 23.6 min). Compounds **2** (8.0 mg) and **3** (6.0 mg) were obtained from sub-fraction C_5_-2 which purified successively on Sephadex LH-20 column (MeOH) and semi-preparative HPLC chromatography (MeOH-H_2_O 38%, v/v, flow rate 3 mL/min; *t*_R_-**3** = 37.3 min, *t*_R_-**2** = 50 min). 

### 3.4. Characterization of Compounds **1**–**3**

Compound **1**: white powder, mp 290–293 °C, ${\left[\alpha \right]}_{D}^{25}$ −44.8 (*c* 0.9, MeOH); IR (KBr) υ_max_ 3342, 2951, 1769, 1740, 1727, 1704, 1660, 1176, 1035 cm^−1^; ^1^H-NMR (400 MHz, C_5_D_5_N) and ^1^^3^C-NMR (100 MHz, C_5_D_5_N) spectroscopic data, see Table 1; positive ion ESIMS *m/z*: 311 [M+H]^+^, 333 [M+Na]^+^; negative ESIMS *m/z*: 655 [2M+Cl]^−^; HRESIMS *m/z*: 311.1852 [M+H]^+^ (calcd for C_17_H_27_O_5_, 311.1853).

Compound **2**: white powder, mp 305–307 °C, ${\left[\alpha \right]}_{D}^{25}$ +33.2 (*c* 0.5, MeOH); IR (KBr) υ_max_ 3392, 2921, 2850, 1754, 1688, 1458, 1384, 1229, 1176 cm^−1^; ^1^H-NMR (400 MHz, MeOD) and ^1^^3^C-NMR (100 MHz, MeOD) spectroscopic data, see Table 2; positive ion ESIMS *m/z*: 413 [M+H]^+^, 435 [M+Na]^+^; negative ESIMS *m/z*: 411 [M−H]^−^, 859 [2M+Cl]^−^; HRESIMS *m/z*: 435.1971 [M+Na]^+^ (calcd for C_21_H_32_O_8_Na, 435.1989).

Compound **3**: yellowish syrup, ${\left[\alpha \right]}_{D}^{25}$ −37.7 (*c* 1.1, MeOH); IR (KBr) υ_max_ 3400, 2923, 2870, 1702, 1458, 1380, 1221 cm^−1^; ^1^H-NMR (400 MHz, C_5_D_5_N) and ^1^^3^C-NMR (100 MHz, C_5_D_5_N) spectroscopic data, see Table 2; positive ion ESIMS *m/z*: 451 [M+Na]^+^, 879 [2M+Na]^+^; negative ESIMS *m/z*: 427 [M−H]^−^, 463 [M+Cl]^−^; HRESIMS *m/z*: 451.1935 [M+Na] ^+^ (calcd for C_21_H_32_O_9_Na, 451.1939).

### 3.5. Determination of the Configurations of Sugar Unit in **2** and **3**

Compounds **2** (2.0 mg) in 1 N HCl (5 mL, 1,4-dioxane–H_2_O, 1:1) was heated under reflux for 8 h. After removal of the solvent, the residue was partitioned between Et_2_O and H_2_O. The water layer was neutralized with 5% NaOH and desalted (Sephadex LH-20, MeOH) to afford the sugar residue (1.0 mg). The sugar residue was derivatized with Sigma Sil-A for 35 min at 70 °C and analyzed by GC-MS [HP-5MS column (30 m × 0.25 mm, 0.25 mm); injection temperature: 250.0 °C; column flow: 1.33 mL/min; ion source temperature: 200.0 °C; interface temperature: 220.0 °C]. In the acid hydrolysate of **2**, d-Glucose was confirmed by comparison of the retention times of the aforementioned derivative with the authentic d-glucose derivative prepared in a similar way, both of which showed identical retention time of 12.17 min. By the same method, the sugar moiety of **3** was identified as d-glucose.

### 3.6. Cytotoxicity Assays

The cytotoxic activity of each compound against human promyelocytic leukemia HL-60 cell was examined *in vitro* at the Second Xiangya Hospital of Central South University, and was determined by the MTT assay [20,21].

## 4. Conclusions

The phytochemical investigation of the aerial parts of *A. artemisiifolia* afforded a new pseudoguaianolide (**1**), two new guaiane-type sesquiterpene glucosides (**2** and **3**), as well as two known sesquiterpene dilactones psilostachyin B (**4**) and psilostachyin (**5**). Although all the isolated compounds were inactive against human promyelocytic leukemia HL-60 cell, the discovery of these new compounds further expands our knowledge of the structural diversity of the sesquiterpenoids produced by the plant *A. artemisiifolia*.

## Supplementary Materials

Supplementary materials can be accessed at: http://www.mdpi.com/1420-3049/20/03/4450/s1.
