# Supplementary material for: New Sesquiterpenoids from Ambrosia artemisiifolia L
Source: Molecules. 2015 Mar 10;20(3):4450–9. doi: 10.3390/molecules20034450 (PMC6272762; doi:10.3390/molecules20034450)
Supplement: Supplementary file 1 [file molecules-20-04450-s001.pdf]

Supporting Information of Compounds 1–3

Figure List:

Figure S1. HR-ESIMS of compound **1**
Figure S2. ^{1}H-NMR of compound **1**
Figure S3. ^{13}C-NMR of compound **1**
Figure S4. ^{1}H-^{1}H COSY of compound **1**
Figure S5. HSQC of compound **1**
Figure S6. HMBC of compound **1**
Figure S7. NOESY of compound **1**
Figure S8. HR-ESIMS of compound **2**
Figure S9. ^{1}H-NMR of compound **2**
Figure S10. ^{13}C-NMR of compound **2**
Figure S11. ^{1}H-^{1}H COSY of compound **2**
Figure S12. HSQC of compound **2**
Figure S13. HMBC of compound 2
Figure S14. NOESY of compound **2**
Figure S15. HR-ESIMS of compound **3**
Figure S16. ^{1}H-NMR of compound **3**
Figure S17. ^{13}C-NMR of compound **3**
Figure S18. ^{1}H-^{1}H COSY of compound **3**
Figure S19. HSQC of compound **3**
Figure S20. HMBC of compound **3**
Figure S21. NOESY of compound **3**

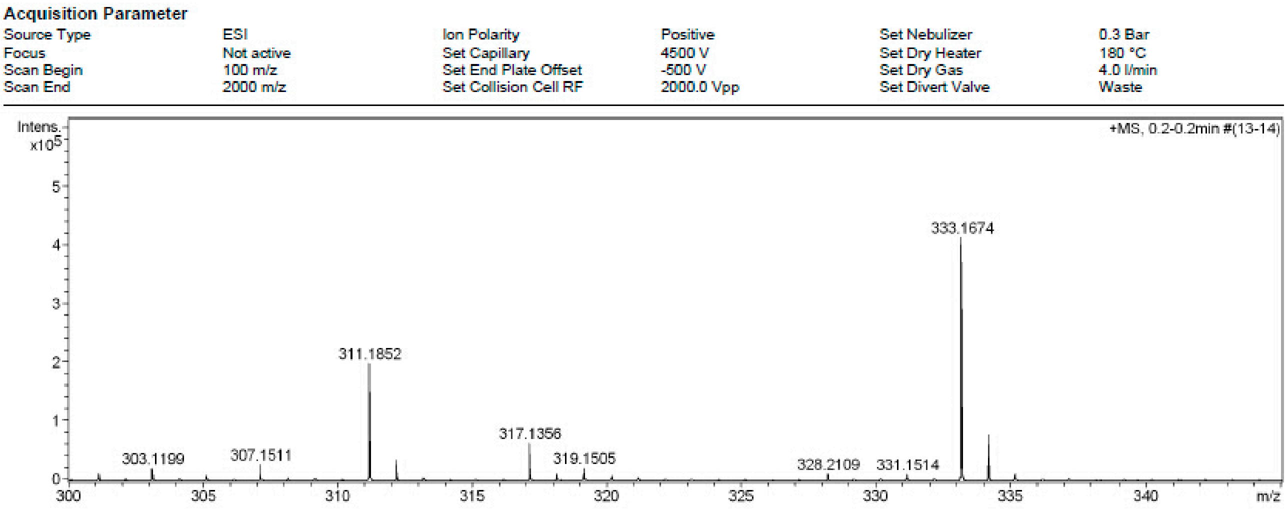

Figure S1. HR-ESIMS of compound **1**.

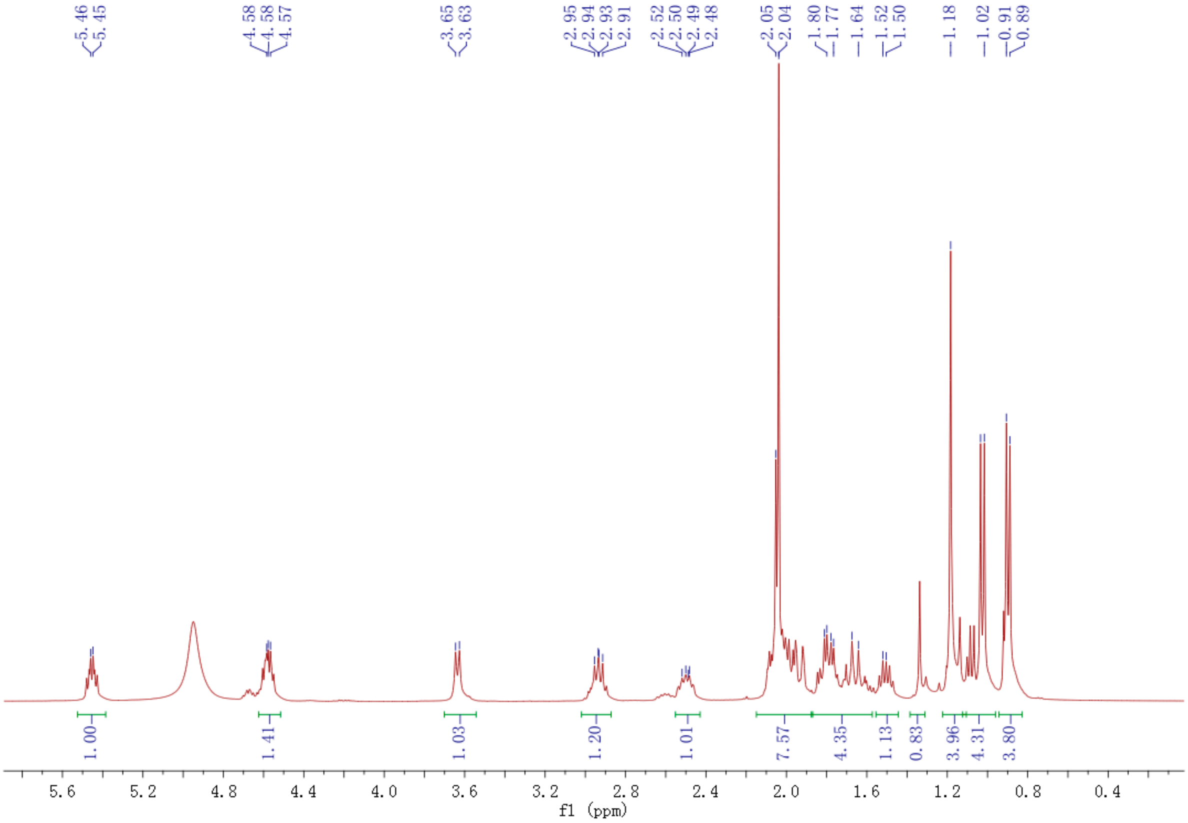

Figure S2. ^{1}H-NMR of compound **1**.

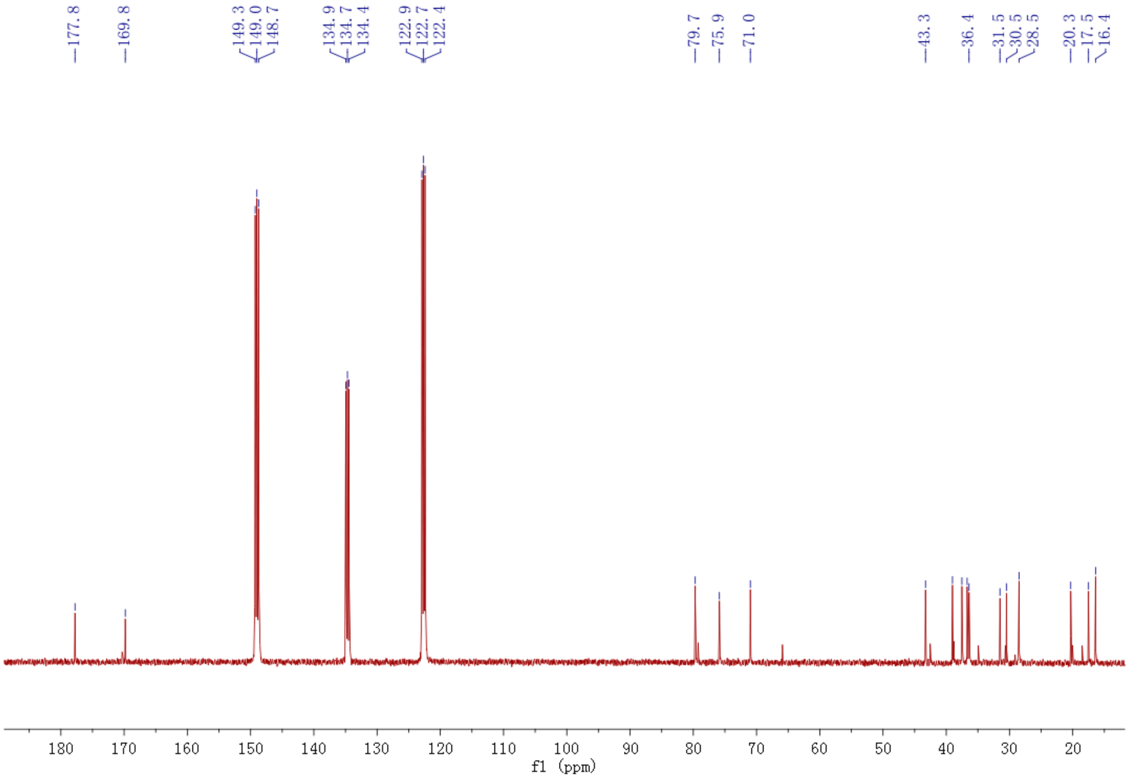

Figure S3. ^{13}C-NMR of compound **1**.

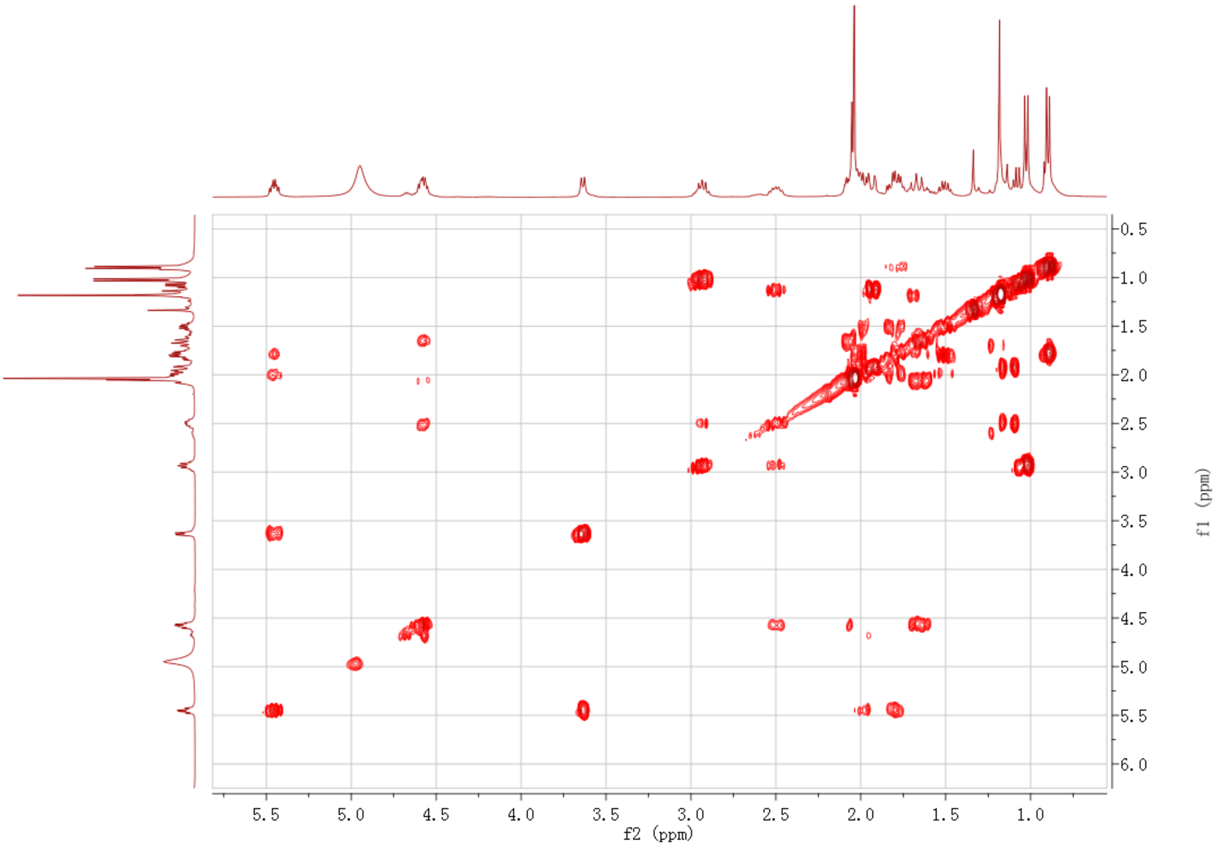

Figure S4. ^{1}H-^{1}H COSY of compound **1**.

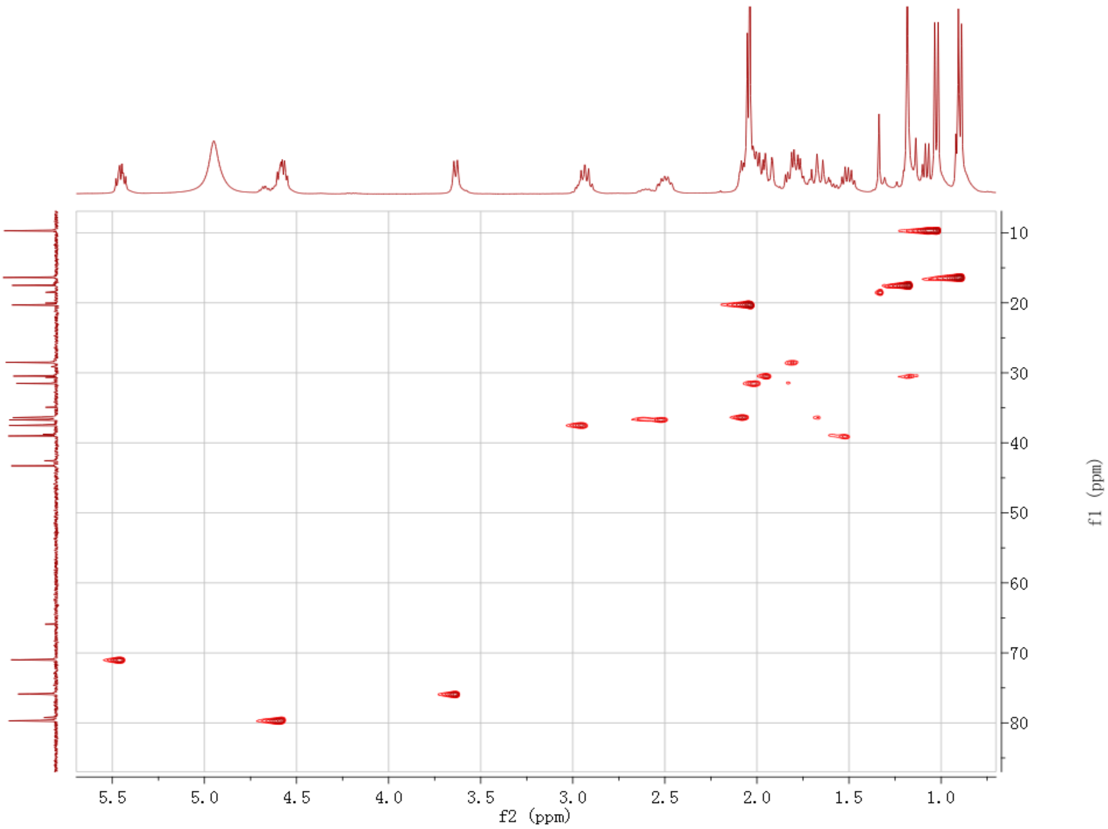

Figure S5. HSQC of compound **1**.

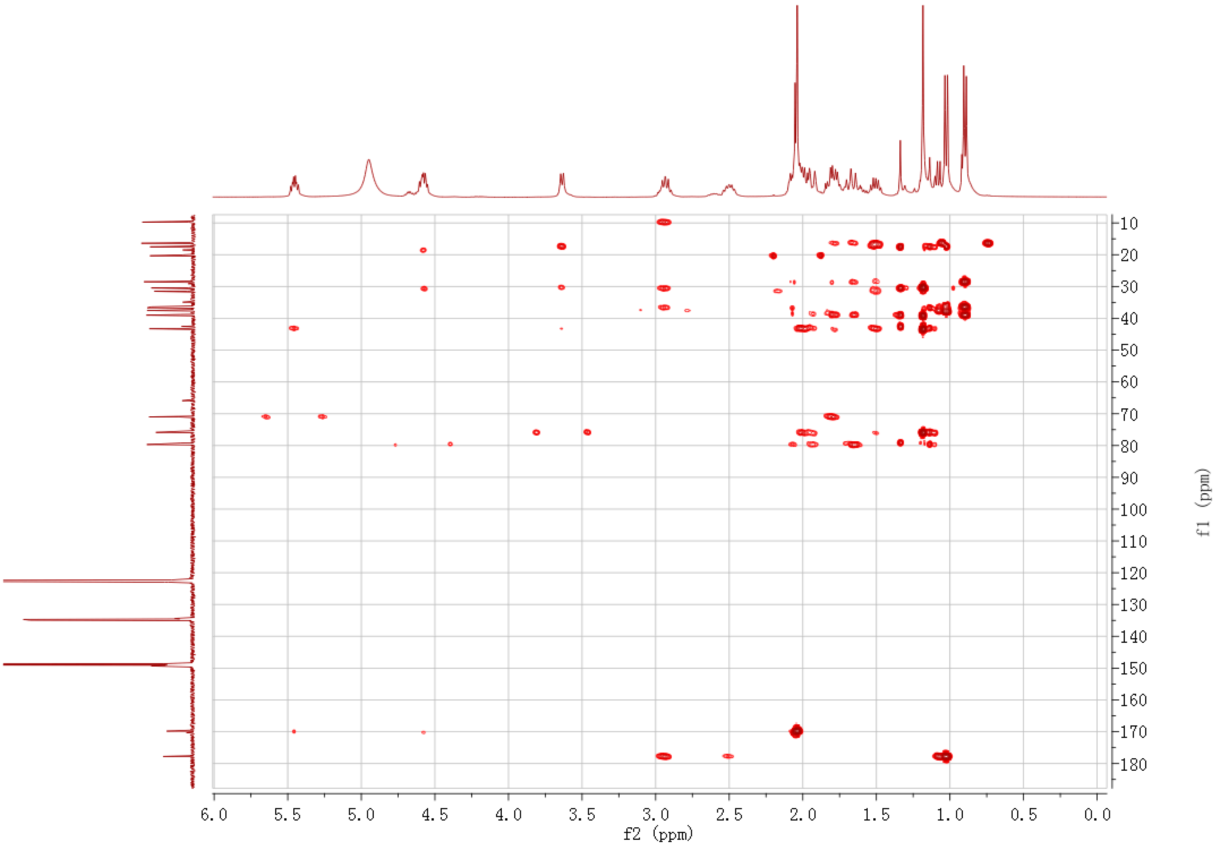

Figure S6. HMBC of compound **1**.

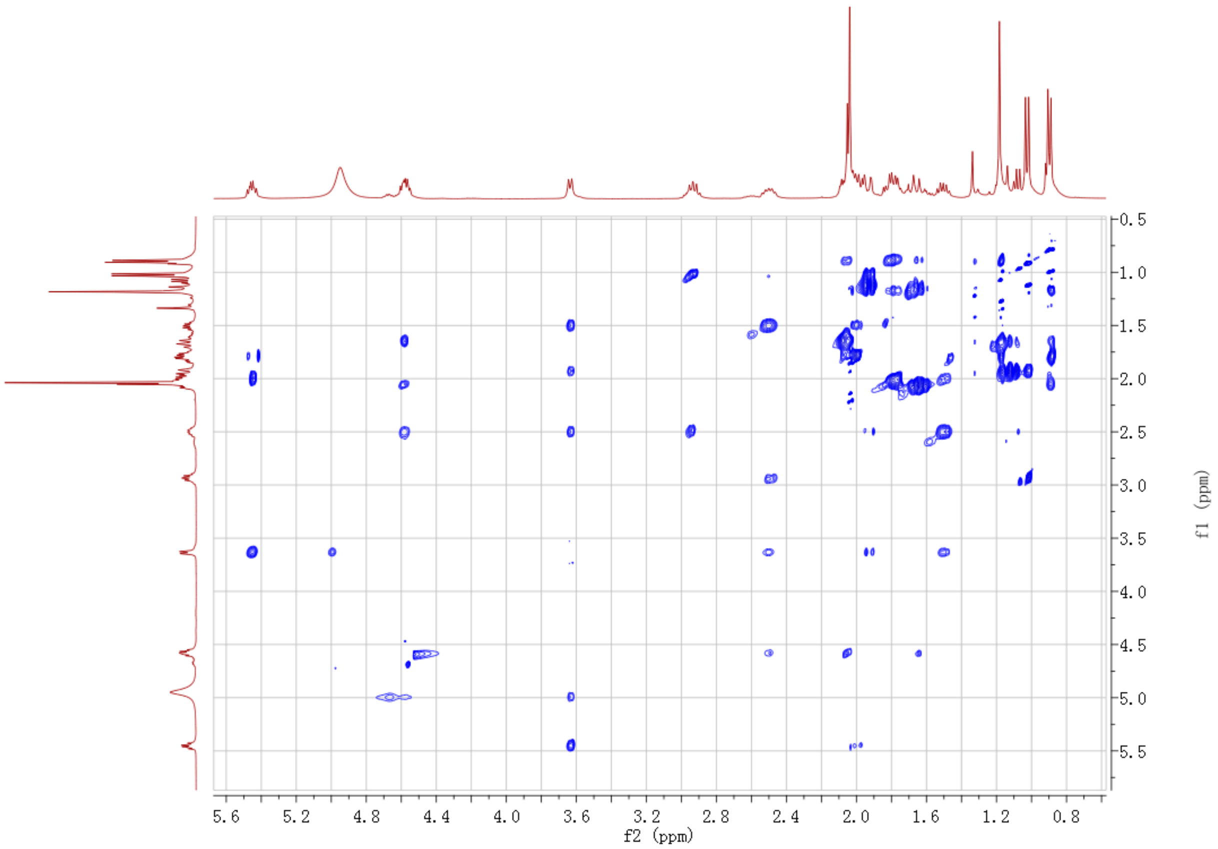

Figure S7. NOESY of compound **1**.

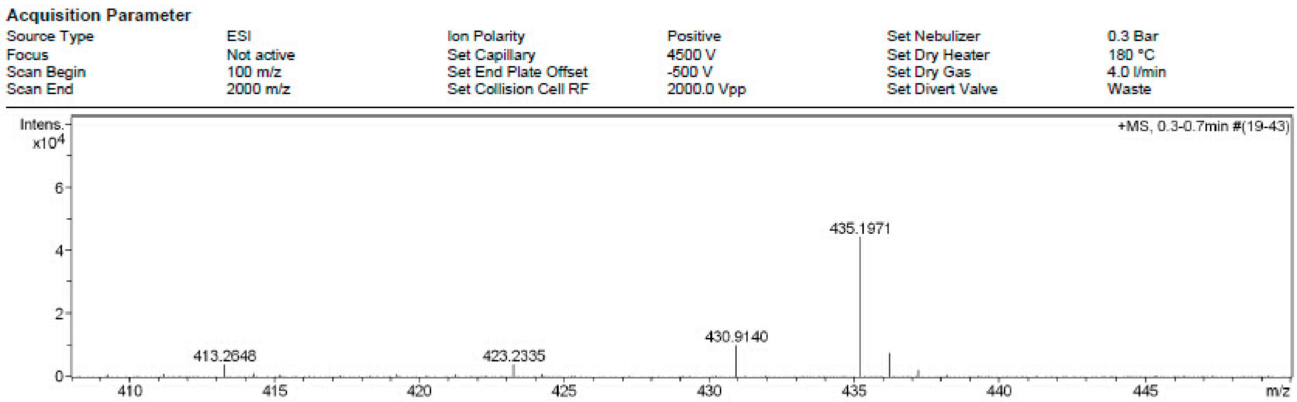

Figure S8. HR-ESIMS of compound **2**.

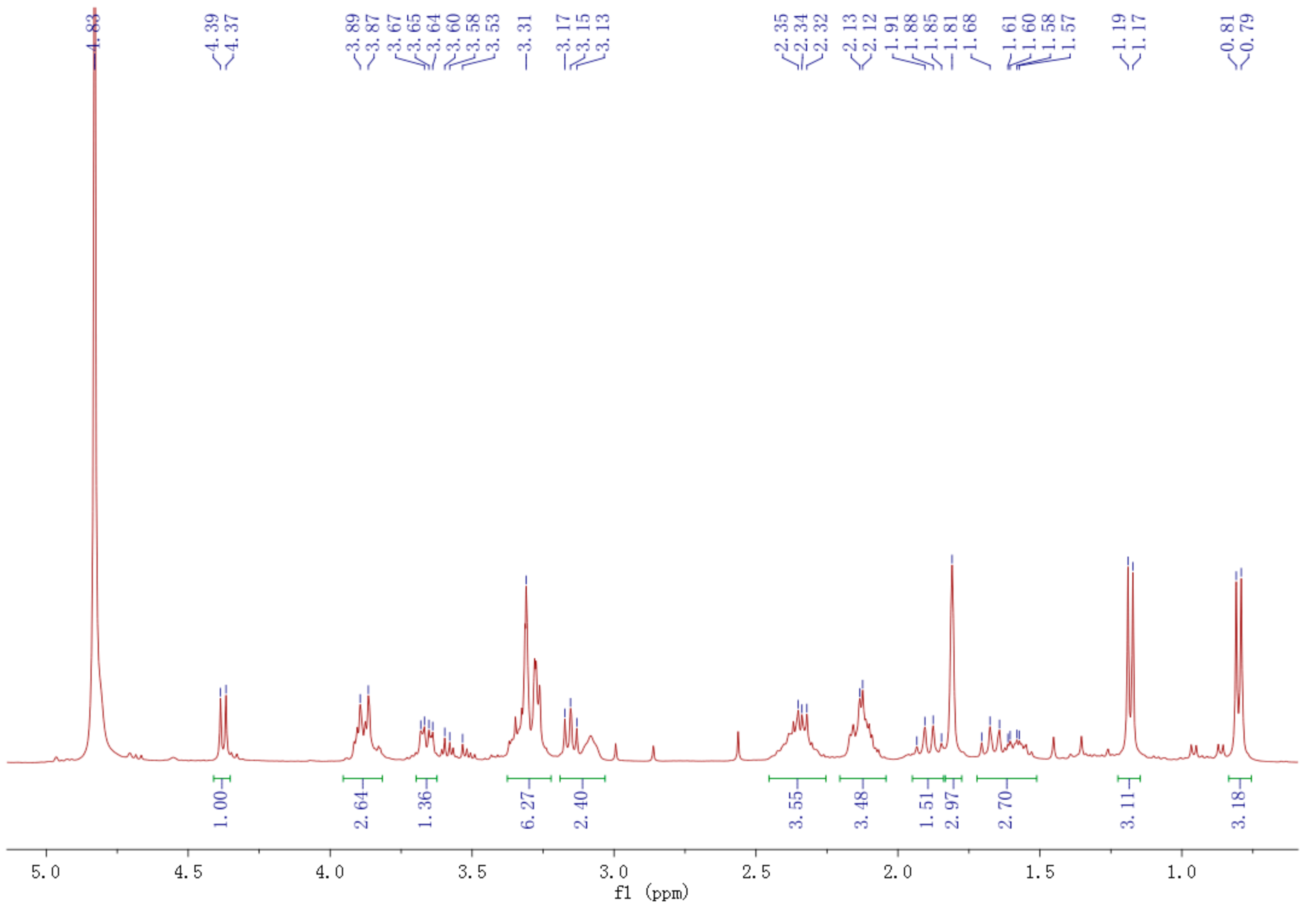

Figure S9. ^{1}H-NMR of compound **2**.

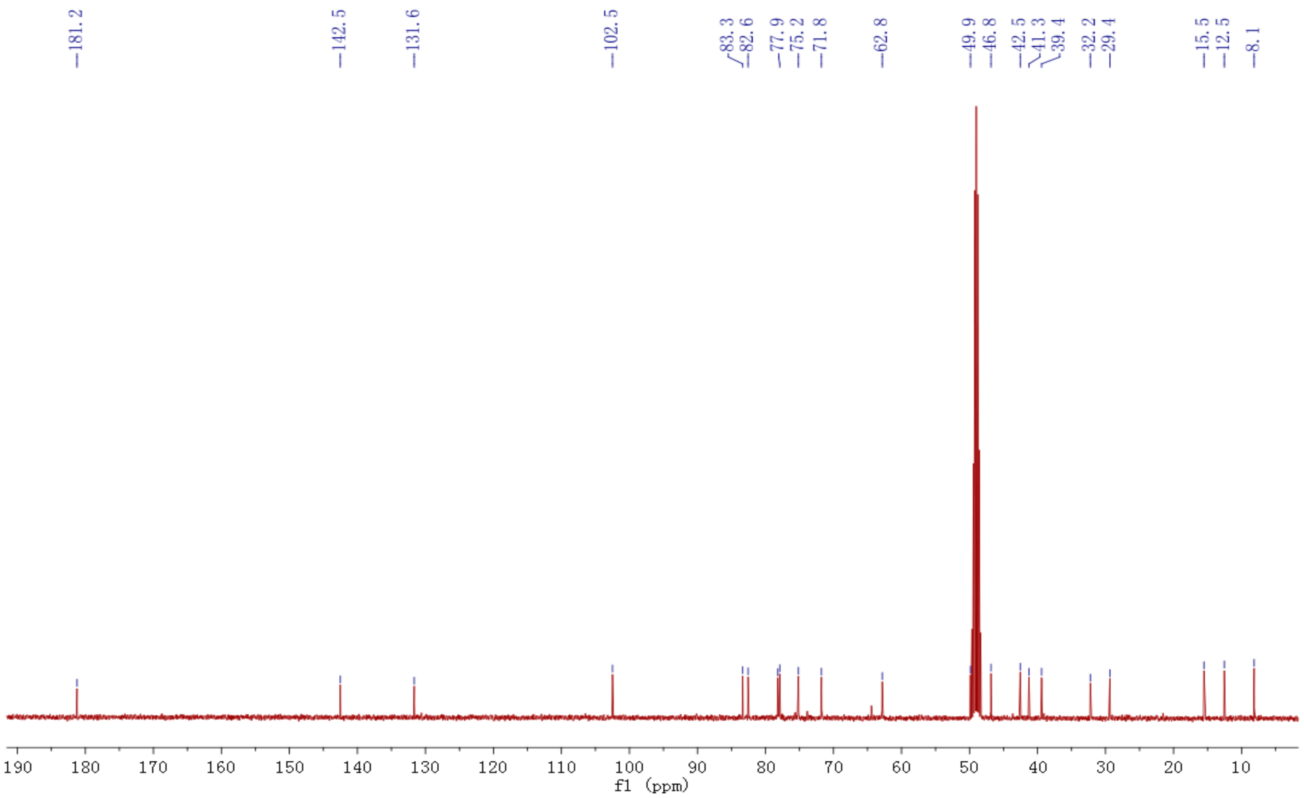

Figure S10. ^{13}C-NMR of compound **2**.

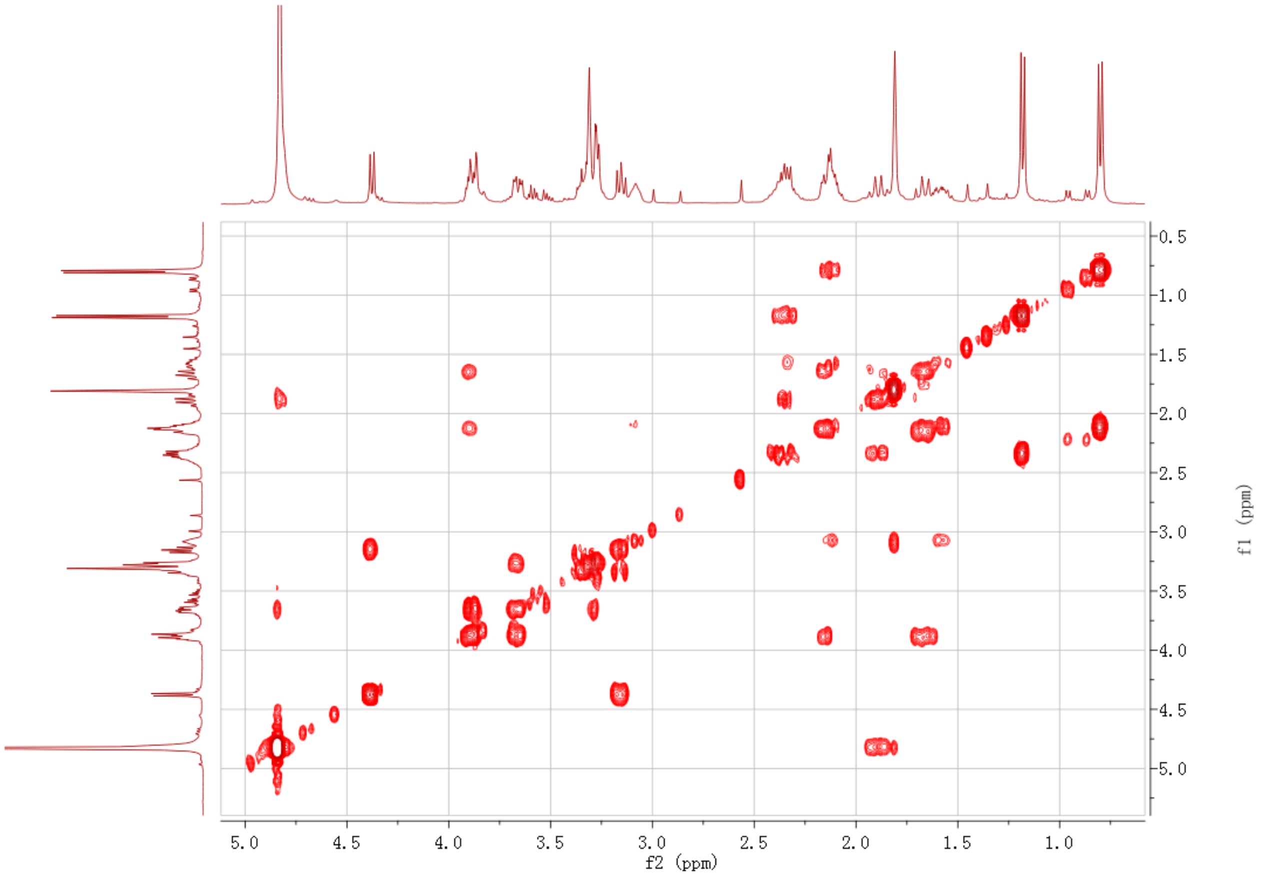

Figure S11. ^{1}H-^{1}H COSY of compound **2**.

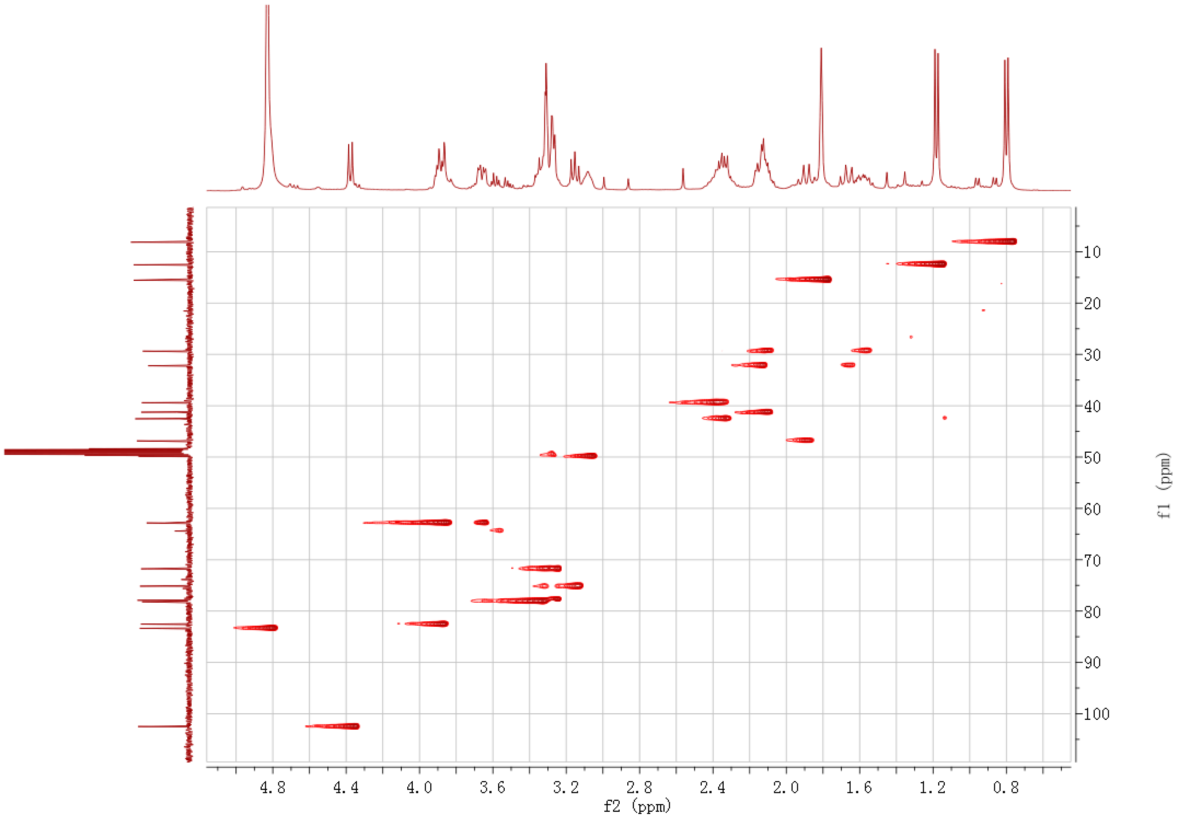

Figure S12. HSQC of compound **2**.

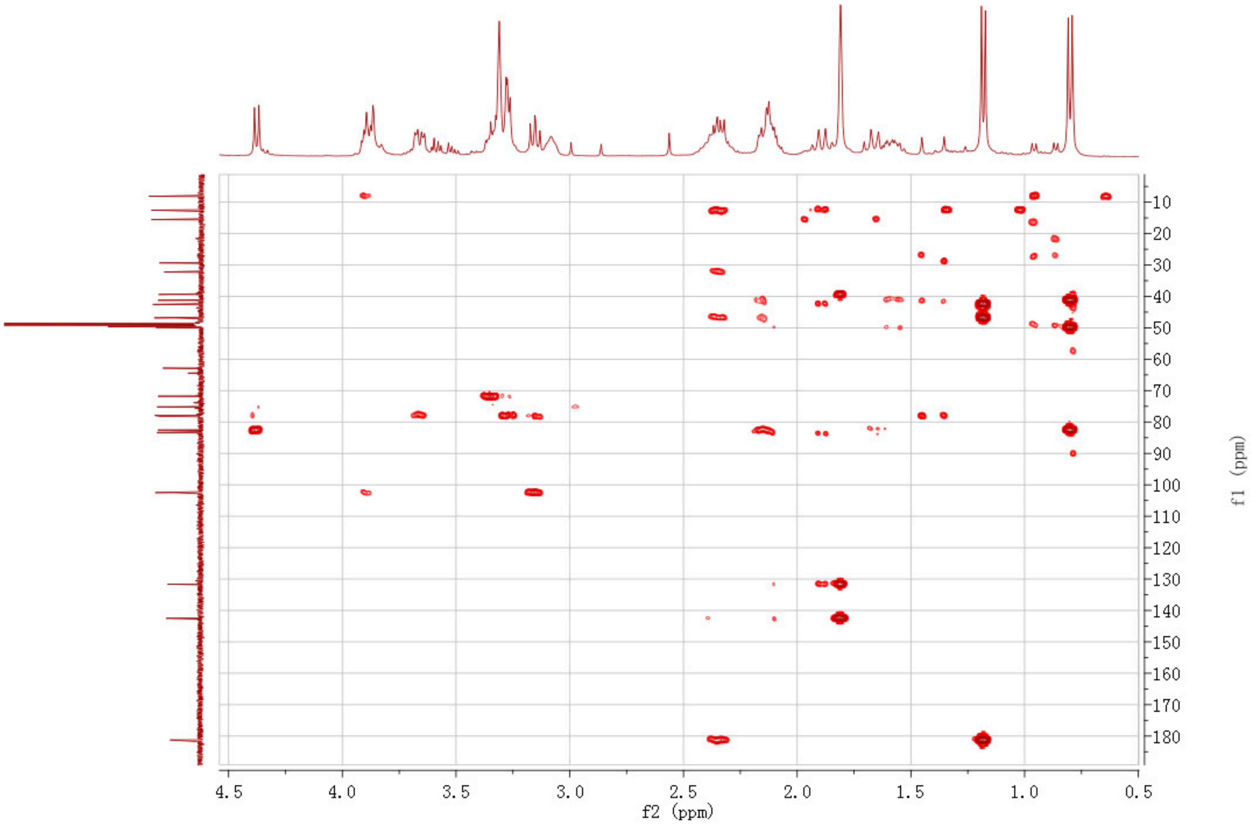

Figure S13. HMBC of compound **2**.

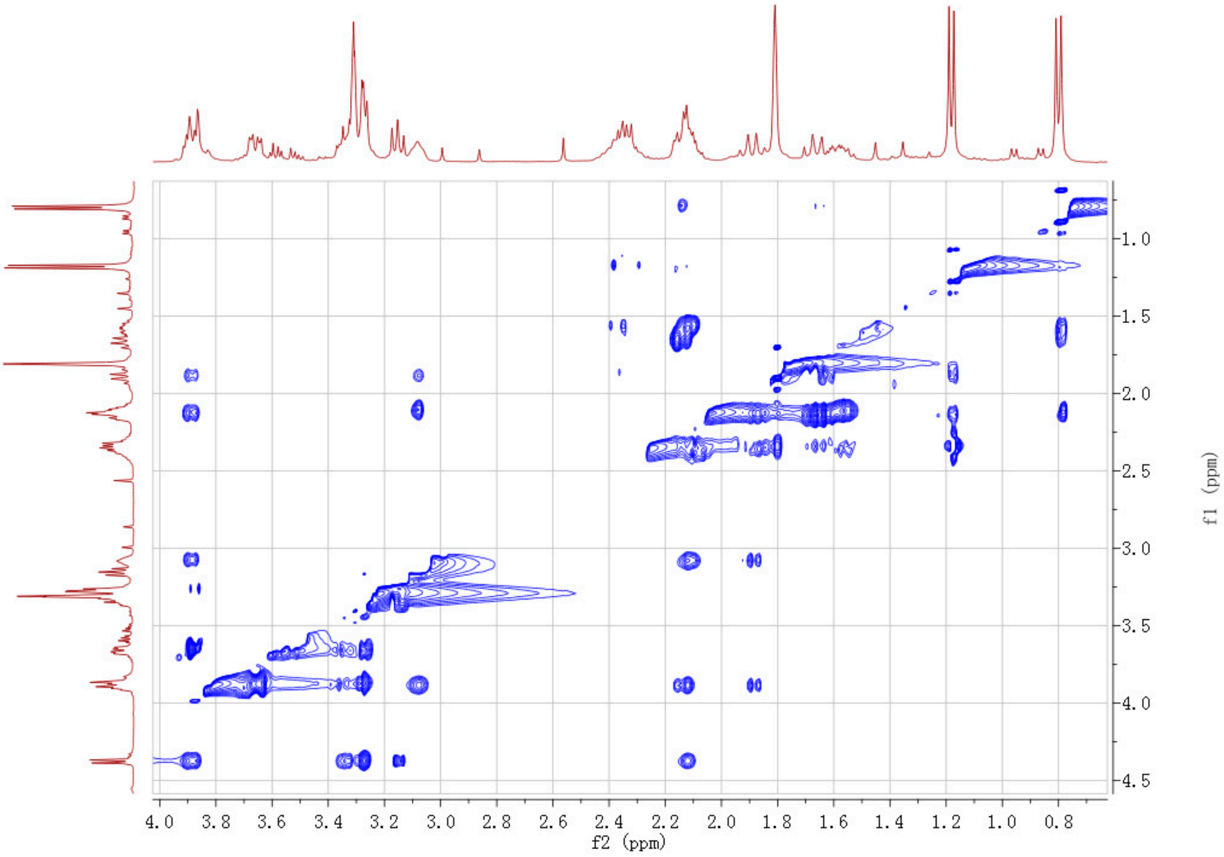

Figure S14. NOESY of compound **2**.

Acquisition Parameter

Source Type	ESI	Ion Polarity	Positive	Set Nebulizer	0.3 Bar
Focus	Not active	Set Capillary	4500 V	Set Dry Heater	180 °C
Scan Begin	100 m/z	Set End Plate Offset	-500 V	Set Dry Gas	4.0 l/min
Scan End	2000 m/z	Set Collision Cell RF	2000.0 Vpp	Set Divert Valve	Waste

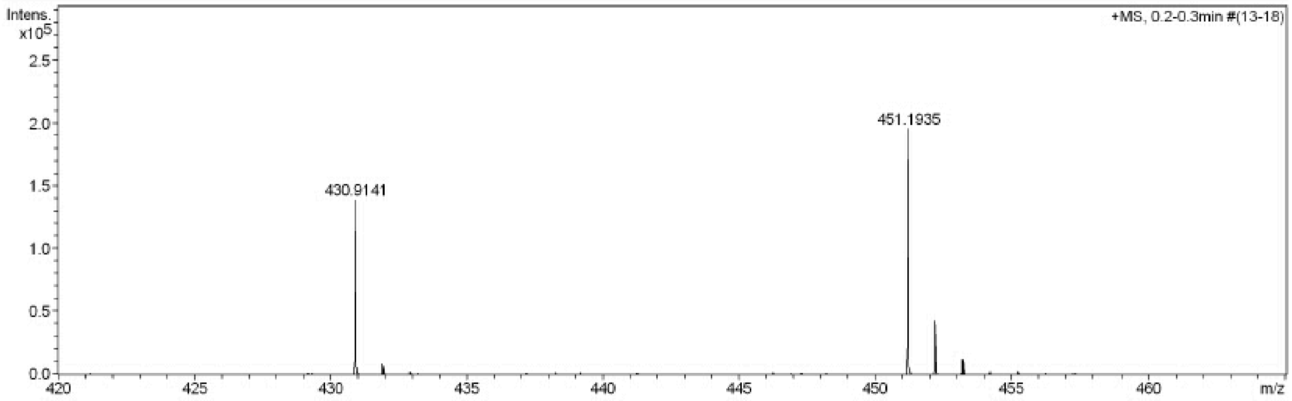

Figure S15. HR-ESIMS of compound **3**.

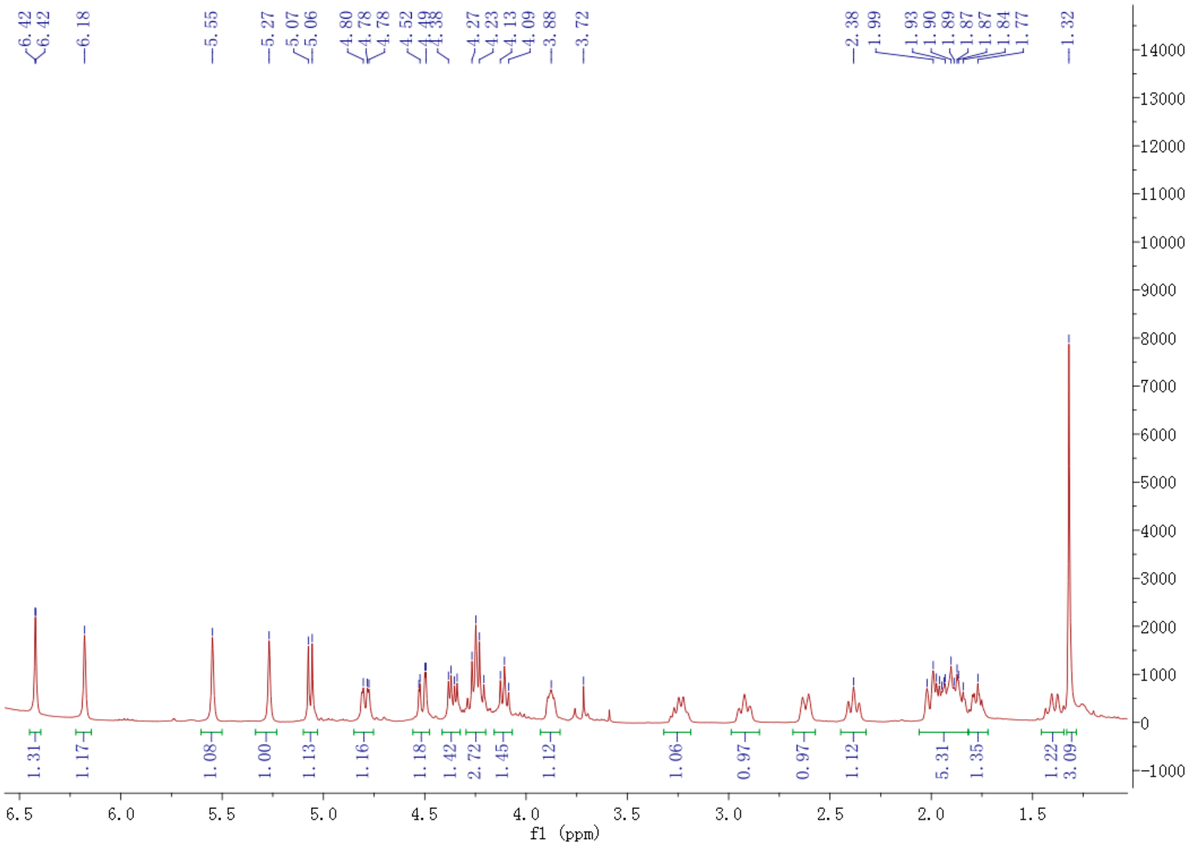

Figure S16. ^{1}H-NMR of compound **3**.

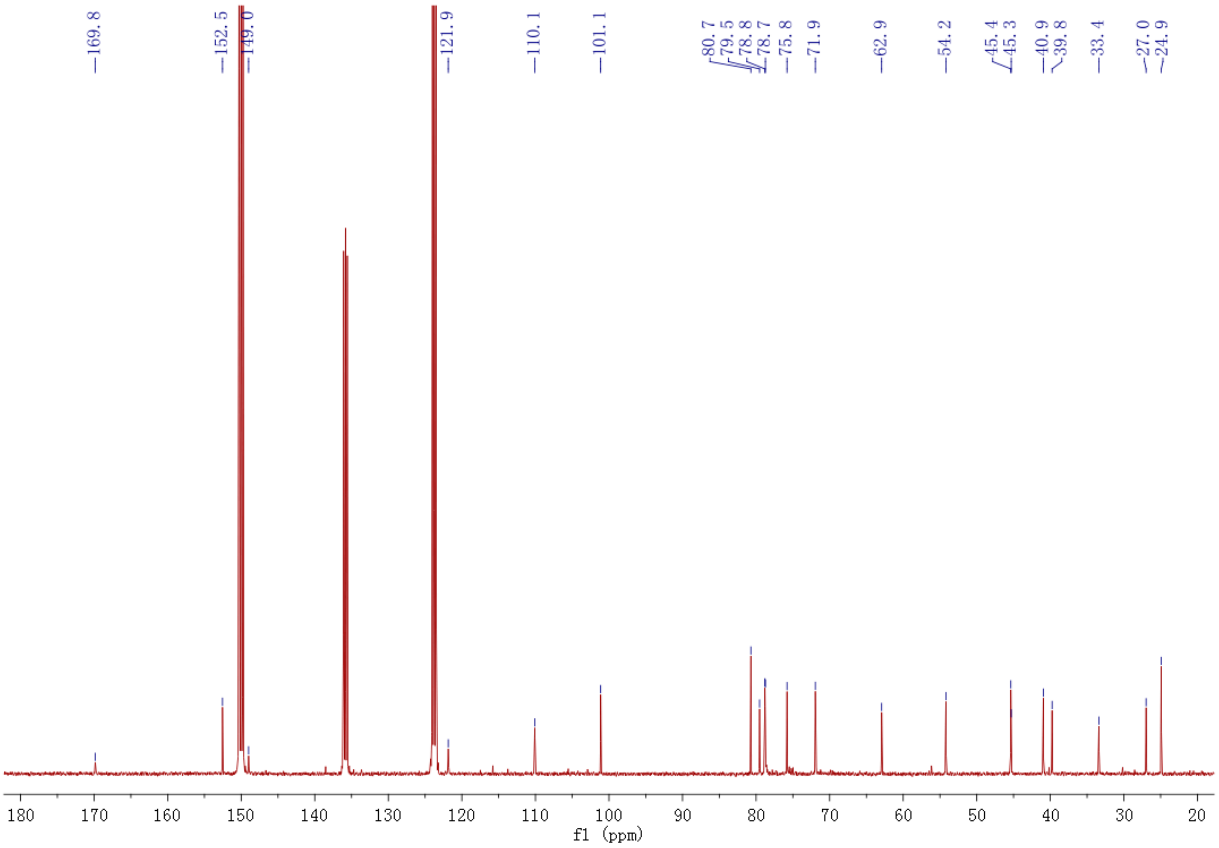

Figure S17. ^{13}C-NMR of compound **3**.

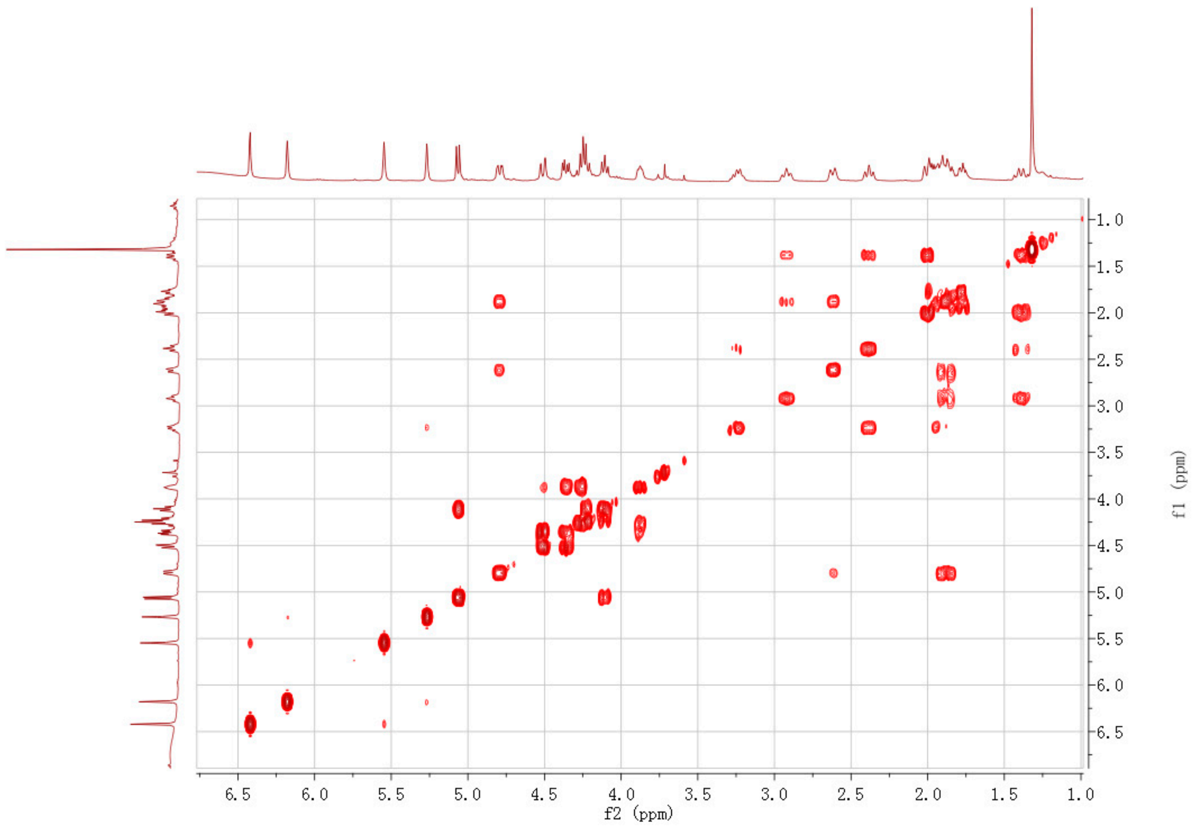

Figure S18. ^1H-^1H COSY of compound **3**.

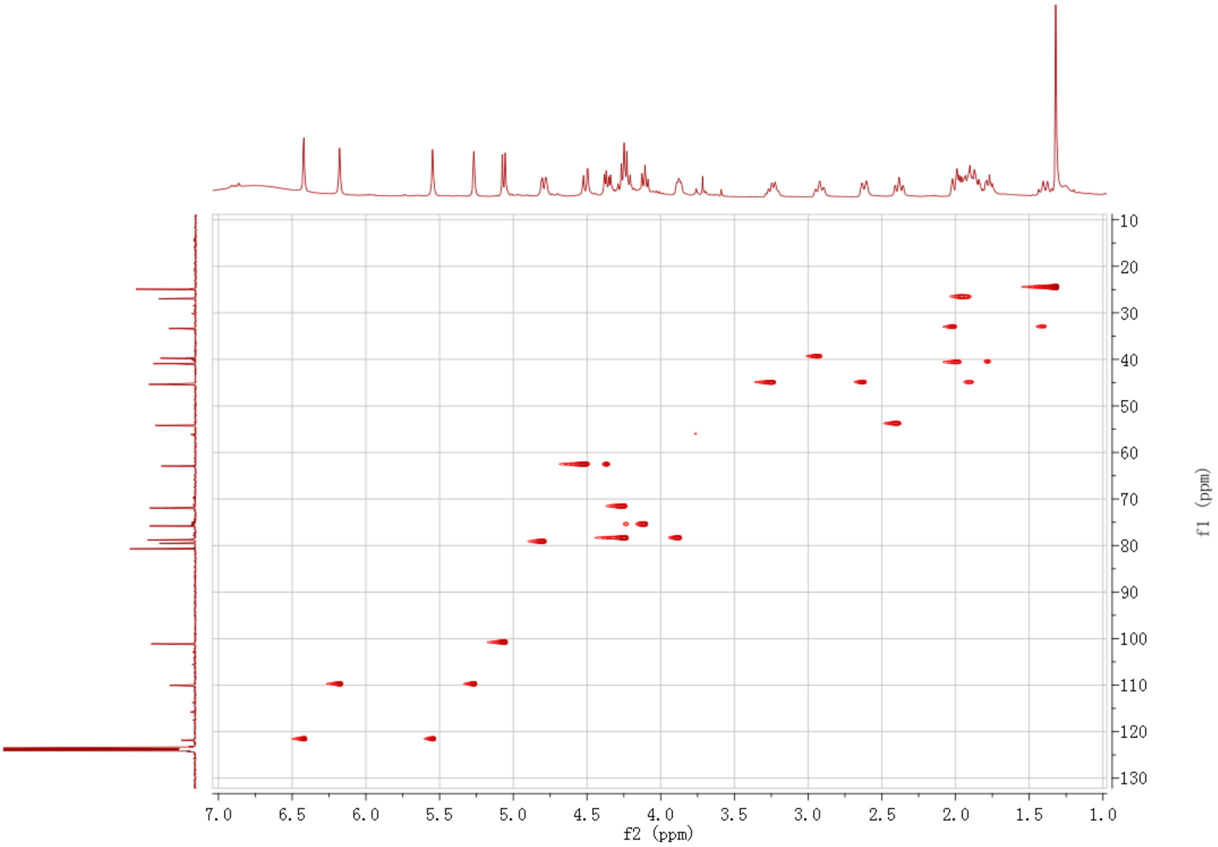

Figure S19. HSQC of compound **3**.

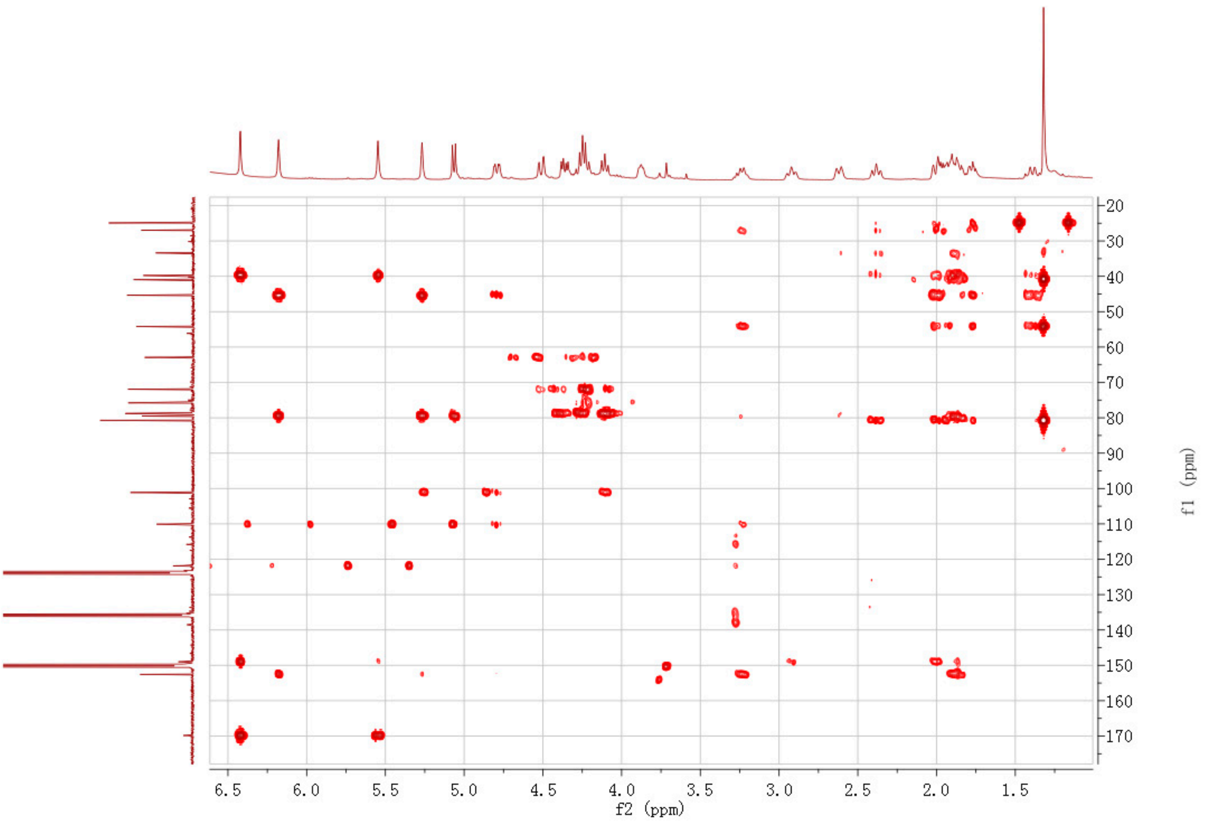

Figure S20. HMBC of compound **3**.

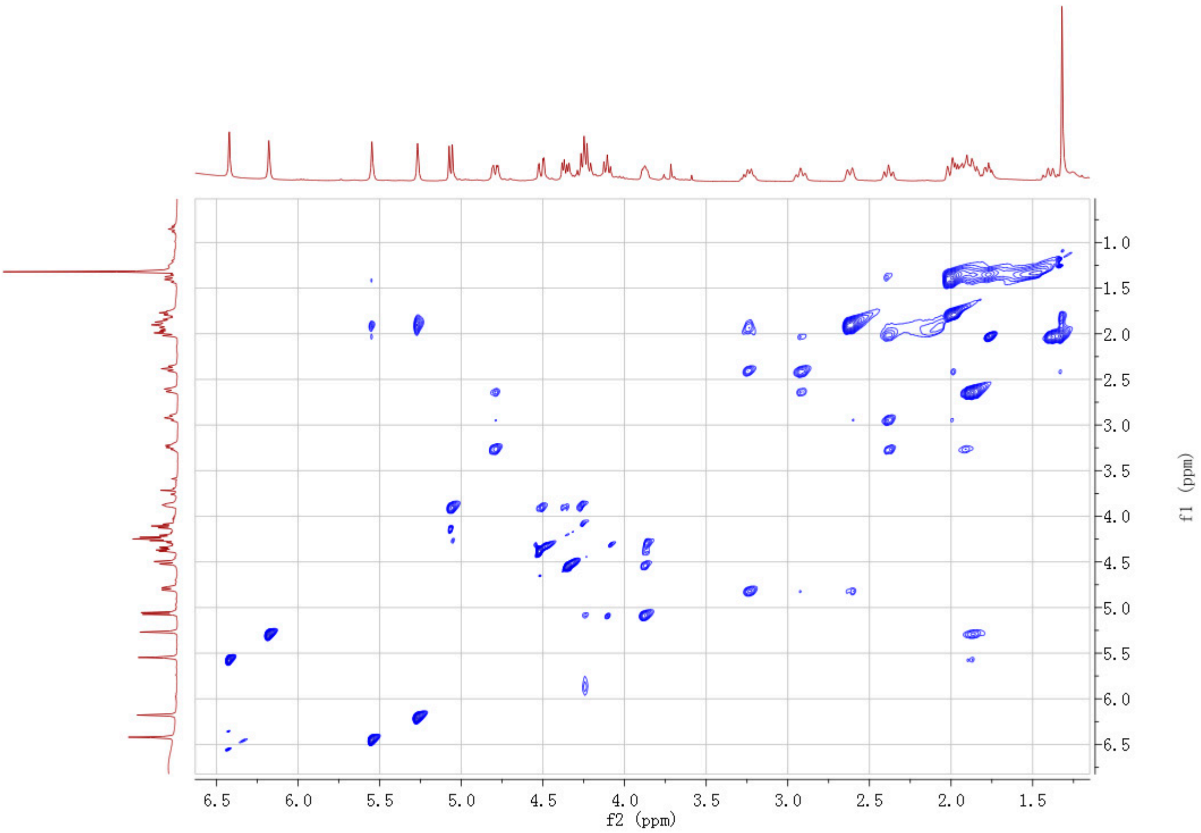

Figure S21. NOESY of compound **3**.
